# Cell cycle regulation by the bacterial nucleoid

**DOI:** 10.1016/j.mib.2014.09.020

**Published:** 2014-12

**Authors:** David William Adams, Ling Juan Wu, Jeff Errington

**Affiliations:** Centre for Bacterial Cell Biology, Baddiley-Clark Building, Medical School, Newcastle University, Richardson Road, Newcastle Upon Tyne, NE2 4AX, United Kingdom

## Abstract

Division site selection presents a fundamental challenge to all organisms. Bacterial cells are small and the chromosome (nucleoid) often fills most of the cell volume. Thus, in order to maximise fitness and avoid damaging the genetic material, cell division must be tightly co-ordinated with chromosome replication and segregation. To achieve this, bacteria employ a number of different mechanisms to regulate division site selection. One such mechanism, termed nucleoid occlusion, allows the nucleoid to protect itself by acting as a template for nucleoid occlusion factors, which prevent Z-ring assembly over the DNA. These factors are sequence-specific DNA-binding proteins that exploit the precise organisation of the nucleoid, allowing them to act as both spatial and temporal regulators of bacterial cell division. The identification of proteins responsible for this process has provided a molecular understanding of nucleoid occlusion but it has also prompted the realisation that substantial levels of redundancy exist between the diverse systems that bacteria employ to ensure that division occurs in the right place, at the right time.

## Highlights

• Nucleoid occlusion prevents cell division over the bacterial chromosome.
• Nucleoid occlusion factors identified in *B. subtilis*, *E. coli* and *S. aureus*.
• Noc and SlmA are sequence specific DNA-binding proteins.
• They both act as spatial and temporal regulators of cell division.
• Using some basic general principles bacteria employ diverse regulatory mechanisms.

**Current Opinion in Microbiology** 2014, **22**:94–101

This review comes from a themed issue on **Growth and development: prokaryotes**

Edited by **Frédéric Boccard**

For a complete overview see the Issue and the Editorial

Available online 17th October 2014

**http://dx.doi.org/10.1016/j.mib.2014.09.020**

1369-5274/© 2014 The Authors. Published by Elsevier Ltd. This is an open access article under the CC BY license (http://creativecommons.org/licenses/by/3.0/).

## Introduction

**Figure 1:**
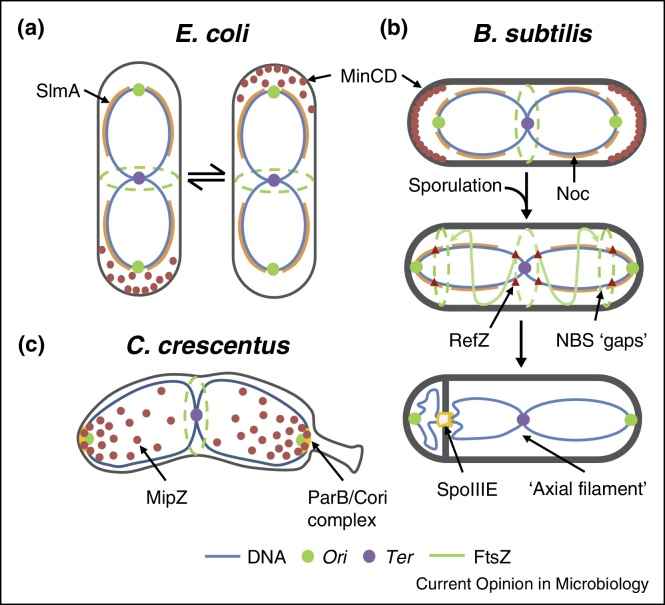
Mechanisms regulating division site selection in rod-shaped bacteria. (**a**–**c**) Schematic cartoons illustrating the general mechanisms regulating division site selection in several well-studied model organisms. (**a**) and (**b**) SlmA in *E. coli* and Noc in *B. subtilis* antagonise Z-ring assembly (dashed green lines) over the DNA using the distribution of their binding sites on the chromosome as a template to guide their activity. MinCD prevents cell division at the poles. In *E. coli* MinCD oscillates from pole to pole (**a**) whereas in *B. subtilis* (**b**) it associates simultaneously with both cell poles. (**b**) During sporulation the nucleoid adopts an elongated configuration—the ‘axial filament’—and Z-rings form at both cell poles. The redistribution of FtsZ from mid-cell to the poles may be promoted by RefZ. Maturation of one polar Z-ring leads to asymmetric cell division, trapping ∼1/3 of the chromosome beneath the closing septum. SpoIIIE then translocates the remaining DNA into the small prespore compartment, allowing spore development to continue. (**c**) In *C. crescentus* the FtsZ inhibitor MipZ uses both the cell poles and the nucleoid as markers to guide its activity, creating a gradient that emanates outwards from the cell poles.

How cell division is coordinated with the replication and segregation of chromosomes is a fundamental problem in biology. Bacteria are no exception. They employ sophisticated regulatory mechanisms to maximise the fitness of progeny by ensuring that they are suitably sized and inherit an intact copy of the genome. Bacteria typically contain a single circular chromosome that is replicated bi-directionally from a single origin of replication (*oriC*; 0°). During replication the newly synthesised sister chromosomes rapidly segregate ‘origin-first’ in opposite directions, before the replication forks rendezvous and terminate in the terminus region (*Ter*; 180°). Once chromosome replication and segregation are complete the cell is ready to divide. In Bacteria this normally occurs by binary fission and in almost all species this is initiated by the assembly of the tubulin homologue FtsZ into a ring-like structure (‘Z-ring’) at the nascent division site (Figure 1) [1]. The Z-ring then functions as a dynamic platform for assembly of the division machinery [2, 3]. Its central role in division also allows FtsZ to serve as a regulatory hub for the majority of regulatory proteins identified to date [2, 4]. Nevertheless, the precise ultra-structure of the Z-ring and whether or not it plays a direct role in force-generation during division remains controversial [5, 6].

Although outwardly a simple process, the division site must be chosen carefully. Division at the pole would produce a non-viable anucleate ‘mini-cell’. Conversely, division through the nucleoid would be catastrophic, generating at least one non-viable cell. In the best studied Gram-positive and Gram-negative rod-shaped model organisms, *Bacillus subtilis* and *Escherichia coli*, the division site is placed precisely at mid-cell [7, 8, 9]. Thus, division results in the production of two equally sized daughter cells. Such remarkable levels of precision are thought to result largely from the combined action of two negative regulatory systems. In the first, the Min system prevents division close to the cell poles, by inhibiting Z-ring assembly [10]. This is accomplished by the FtsZ inhibitor MinC, which is recruited into a membrane-associated complex by the ParA-like ATPase MinD [11]. In *E. coli* MinCD oscillates from pole-to-pole [12, 13, 14] whereas in *B. subtilis* it is recruited to both cell poles [15], but the net result is the same, with the active complex enriched at the cell poles (Figure 1a,b).

The second regulatory system involves the long-standing observation that the nucleoid (bacterial chromosome) can itself act as a cell cycle ‘checkpoint’ and prevent division until the replicated sister chromosomes have segregated—a process termed nucleoid occlusion [16, 17, 18]. The foremost role of this process is in the ‘anti-guillotine’ checkpoint, which prevents catastrophic bisection of the genome by the division machinery. This is achieved by preventing assembly of the Z-ring over the nucleoid (Figure 1a,b). Consequently, nucleoid occlusion might not only act to protect DNA, but likely also acts positively to help identify the division site by directing the division machinery to the DNA-free zone that develops between the newly replicated chromosomes. Although widely recognised as a potentially critical regulatory system, it was only in the last decade that specific factors involved in this process were identified. Additionally, it is now known that bacterial chromosomes are subject to intricate large-scale organisation, for example, structured macro-domains that occupy specific positions within the cell [19, 20]. Moreover, translation also occurs in a spatially restricted manner [21]. Therefore, besides acting as a ‘template’ for specific regulatory proteins, the overall organisation and activity of the nucleoid may also play a more general role in regulating division. In this review we will describe recent progress in understanding the process of nucleoid occlusion as well as highlighting some of the diverse solutions employed by less-well studied bacteria.

## Specific nucleoid occlusion factors

About 10 years ago the first *n*ucleoid *oc*clusion proteins were identified. Noc in *B. subtilis* [22^••^], and in parallel work, SlmA (*s*ynthetic-*l*ethal with *m*in) in *E. coli* [23^••^]. The absence of these proteins allows cell division to occur over the nucleoid under conditions in which DNA replication or cell division are perturbed [22••, 23••]. Both proteins inhibit division when overproduced and, as might be expected, are synthetic-lethal with defects in the Min system and other genes involved in division site selection; a phenotype that facilitated their initial identification [22••, 23••]. Contrary to expectations, however, the loss of two regulatory systems does not lead to unfettered division. Instead, it causes a severe division block, apparently because FtsZ assembles indiscriminately throughout the cell, such that it is unable to form a productive structure at any one particular site [22••, 23••].

**Figure 2:**
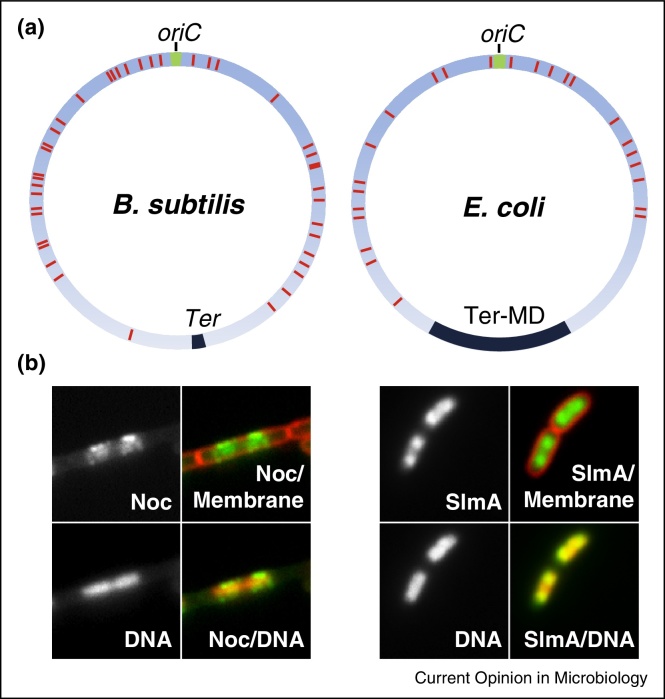
Asymmetric distribution of nucleoid occlusion protein binding sites. (**a**) Distribution of the NBSs in the *B. subtilis* chromosome and the SBSs in the *E. coli* chromosome. The approximate numbers and locations of the sites (red bars) are depicted as described in [24••, 26]. (**b**) Simultaneous localisation of nucleoid occlusion factors (green) Noc (Noc-YFP) in *B. subtilis* (left) and SlmA (GFP-SlmA) in *E. coli* (right) overlaid with both DNA and cell membrane (in red), as indicated. Note the absence of Noc and SlmA from the central regions of the nucleoids.

To function properly nucleoid occlusion factors must act in a controlled manner. An obvious mechanism would be to link their activity to DNA binding. In *B. subtilis* chromatin affinity precipitation followed by microarray analysis (ChAP-Chip) identified around 70 Noc binding sites (NBSs), with a 14 bp palindromic consensus sequence (Figure 2a) [24^••^]. *In vitro* and *in vivo* experiments confirmed that Noc binds specifically to this sequence. Importantly, the introduction of a multi-copy plasmid carrying a single NBS led to a severe division defect, which was dependent on both the ability of Noc to bind DNA and on the presence of the NBS on the plasmid [24^••^]. These findings indicated that Noc activity is coupled to specific DNA binding and are consistent with the idea that the relatively mild division defect caused by Noc overproduction is due to the spatial constraints imposed by the nucleoid. Likewise, in *E. coli* SlmA binds specifically to around 24–52 palindromic SlmA binding sites (SBSs) (Figure 2a) [25, 26]. Importantly, specific DNA binding was also shown to enhance SlmA activity [25, 26].

Strikingly, the NBSs and SBSs are not distributed uniformly throughout the respective chromosomes of *B. subtilis* [24^••^] and *E. coli* [25, 26], in both cases they are noticeably underrepresented in the T*er* regions (Figure 2a,b). Since the *oriC* proximal regions of chromosome are replicated first and are thought to rapidly segregate towards the opposing cell poles, it was proposed that this might allow Noc/SlmA to also act as timing devices, and couple the initiation of cell division to the closing stages of DNA replication/segregation [24••, 25, 26]. This model is strongly supported by the finding that in both organisms introducing an array of NBSs or SBSs into the terminus region leads to delayed cell division [24••, 26].

Despite their remarkably similar roles, Noc and SlmA are totally unrelated and are members of the ParB and TetR DNA binding protein families, respectively [22••, 23••]. Moreover, as discussed below, they appear to act on division by completely different mechanisms.

## SlmA interacts directly with FtsZ to inhibit its polymerisation

**Figure 3:**
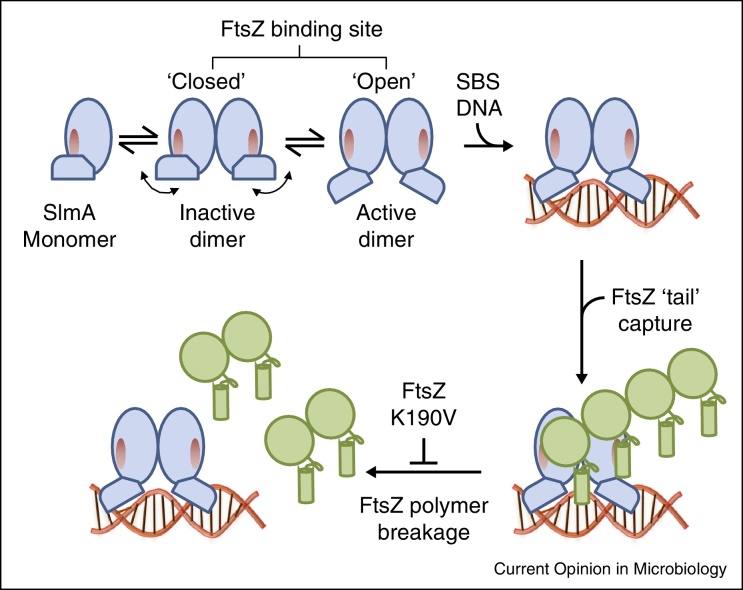
Current model for SlmA mode of action. The SlmA dimer interconverts between inactive and active forms due to flexibility in the HTH-DNA binding domains, which occlude the FtsZ binding sites (red ovals). SBS-DNA binding ‘locks’ the SlmA dimer in the active conformation allowing SlmA to interact with FtsZ polymers (green) via their C-terminal tail. Further interaction(s) between SlmA and FtsZ leads to polymer disassembly, possibly by inducing a conformational change that enhances FtsZ GTPase activity [26, 29••]. FtsZ K190V is a variant that interacts with SlmA but is resistant to its activity [29^••^]. Note that SlmA binds DNA as a dimer of dimers [28^•^] but for simplicity only one is shown, see text for full details. Figure adapted from [27^•^].

A variety of genetic and biochemical experiments suggest that SlmA interacts directly with FtsZ [23••, 25, 26, 27•, 28•]. Recently, Cho and Bernhardt [27^•^] identified the FtsZ binding interface on SlmA using an elegant genetic screen that allowed mutants defective in DNA-binding to be quickly discarded. Interestingly, the FtsZ binding site sits in close proximity to the helix-turn-helix DNA-binding domain, and is partially occluded by it. The authors suggest a simple model in which SBS binding induces a conformational change in the DNA-binding domains, which activates SlmA by revealing an otherwise occluded interface (Figure 3). Importantly, this model has subsequently been corroborated by the determination of the crystal structures of the SlmA-SBS complex, which indicate that DNA-binding locks the flexible HTH domain into a single conformation [28^•^]. However, the precise mechanism by which it prevents Z-ring assembly has remained controversial.

Based on models derived from small angle X-ray scattering (SAXS) and the observation of large ribbons of FtsZ-SlmA-SBS by electron microscopy Tonthat *et al*. [25] proposed that the SlmA dimer facilitates the assembly of non-productive anti-parallel FtsZ filaments on DNA. Conversely, Cho *et al*. [26] presented convincing evidence that SlmA is a DNA-activated antagonist of FtsZ polymerisation and suggested that it likely acts by severing growing FtsZ polymers *in vivo*. This idea is supported by the robust correlation between the ability of SlmA mutants to inhibit FtsZ assembly *in vitro* and mediate nucleoid occlusion *in vivo* [26, 27•]. Moreover, obligate heterodimers of SlmA, in which only one subunit is capable of interacting with FtsZ, are functional for nucleoid occlusion [27^•^], seemingly ruling out the antiparallel filaments mechanism [25].

Recently, however, crystal structures of the SlmA-SBS complexes from *E. coli*, *Klebsiella pneumoniae* and *Vibrio cholerae* have revealed that SlmA binds the SBS as a dimer of dimers [28^•^]. Unusually, it also distorts the DNA either side of the SBS, which might allow the localised binding of additional SlmA molecules. Since SlmA is present at around 3–400 molecules per cell [23^••^] this would allow *c.a.* 4–8 dimers of SlmA per SBS. If multiple dimers of SlmA are also present *in vivo* this could allow nucleation of FtsZ filaments within non-productive structures, and remain compatible with the observation that only one FtsZ binding site per dimer is required for activity. Despite this possibility, such a mechanism now seems unlikely. Du and Lutkenhaus [29^••^] have recently shown that SlmA-SBS inhibits FtsZ polymerisation *in vitro* under all conditions previously tested. Crucially, the identification of FtsZ mutants that are insensitive to this activity even though they can still interact with SlmA-SBS demonstrates unambiguously that SlmA plays an active role in disassembling FtsZ polymers (Figure 3) [29^••^]. Intriguingly, the authors also established that SlmA binds to the conserved C-terminal tail of FtsZ, which acts as an adaptor for the binding of at least five other division proteins [29^••^]. Since the tail itself is not required for FtsZ assembly, how SlmA stimulates polymer disassembly remains an open question.

## Noc associates with both DNA and the cell membrane

Understanding the mechanism by which Noc acts has proved more challenging. In *B. subtilis* Noc is an abundant protein (∼4500 molecules per cell) and forms large nucleoprotein complexes at the NBSs [24^••^]. It localises to the nucleoid and forms dynamic foci at the overlying cell periphery (Figure 2b) [24^••^]. An early hypothesis was that these foci represented sites of interaction between Noc and its target. Nevertheless, all attempts to identify a direct protein target have so far been unsuccessful [24••, 30]. Recently, however, evidence has been discovered that Noc associates directly with the cell membrane and that complex assembly at NBSs controls this activity [Adams, Wu and Errington, unpublished]. Even so, in the absence of a defined target, the question remains—how does Noc inhibit division?

The developmental lifestyle of *B. subtilis* poses additional challenges (Figure 1b). During sporulation the chromosomes adopt an elongated configuration with the *oriC* regions tethered to the poles and the *Ter* regions at mid-cell. An asymmetric septum then forms close to one of the poles, trapping roughly one-third of the chromosome. The remaining DNA is then ‘pumped’ into the prespore compartment by the DNA-translocase SpoIIIE [31]. Thus, Noc activity must be relieved at the cell poles and Z-ring assembly prevented at mid-cell. Noc activity may be attenuated by the down-regulation of *noc* expression [32] coupled with an underrepresentation of NBSs in the regions close to the trapping event (Figure 1b) [24^••^]. Alternatively, the altered chromosome organisation might itself inactivate Noc. Interestingly, the TetR-like protein RefZ (*re*gulator of *F*ts*Z*) was recently shown to promote the redistribution of Z-rings from mid-cell to the cell poles (Figure 1b) [33]. It appears to act on FtsZ directly via a DNA-dependent mechanism, suggesting that other DNA-binding proteins may use the specific conformation of the nucleoid during sporulation to help specify the division site [33].

## Critical role for Noc in *S. aureus*

Since the chromosome occupies the majority of the cytoplasm in coccoid bacteria such as *Staphylococcus aureus*, nucleoid occlusion might be expected to play a more central role, especially given that in many cocci there is no Min system [34]. Indeed, Veiga *et al.* [35^•^] recently showed that the deletion of *noc* results in a significant increase in cell size and strikingly, around 15% of cells contained division septa assembled over the nucleoid. Importantly, the detection of a similar frequency of DNA breaks confirmed that the DNA was bisected by the division machinery and not just trapped by it, thus indicating that even during normal growth conditions Noc plays a critical role [35^•^]. Given its clinical importance it will be interesting to test whether the *noc* mutant is attenuated for virulence. Similar to *B. subtilis*, microscopy suggests that Noc is absent from the terminus region of the *S. aureus* chromosome [35^•^], consistent with the prediction of a highly asymmetric distribution of NBSs [30]. Interestingly, *S. aureus* divides in three consecutive perpendicular planes. Veiga *et al.* [35^•^] propose a model in which segregation of the chromosomes parallel to the incipient division septum provides only one possible plane free from nucleoid occlusion, which in combination with another geometric cue, perhaps a division ‘scar’ [36], then restricts the selection of the next division plane.

## Noc/SlmA independent nucleoid occlusion

In *B. subtilis* or *E. coli* cells lacking both a functional Min and nucleoid occlusion systems FtsZ assembly still exhibits a clear bias towards the inter-nucleoid spaces [22••, 23••, 37•], indicating that there are probably further mechanisms governing division site selection in these organisms. Intriguingly, in *E. coli* cells with unusual shapes the nucleoid appears to be the primary factor influencing division site selection [38]. Likewise, experiments with blocked or non-replicating nucleoids in *B. subtilis* [39] and *E. coli* [40] have shown that the nucleoid prevents Z-ring assembly in its vicinity, independently of both Noc/SlmA and the SOS-response. One possibility is that there are additional, but as yet uncharacterised, nucleoid occlusion factors present. Indeed, some possible candidates exist, though any involvement in division site selection is yet to be determined [41, 42].

Another possibility is that the activity or organisation of the nucleoid may itself play a role in restricting Z-ring assembly [43]. One classical proposal is that the presence of large membrane-associated complexes in the vicinity of the nucleoid resulting from transertion; the coupled transcription, translation and insertion of membrane proteins, generates a short range inhibitor of cell division [16, 17, 18, 44]. The recent demonstration that loci encoding membrane proteins are repositioned towards the membrane upon induction lends support to this idea [45]. Alternatively, specific loci or chromosomal domains may play an active role. MatP, a protein that organises the *Ter* macrodomain (MD) of *E. coli* [46] is recruited to the Z-ring via a direct interaction with ZapB [47^•^]. This could simply serve as a convenient way to retain the *Ter* MD at mid-cell so that it can be processed by FtsK [48, 49, 50]. However, recent work by Bailey *et al*. [51], suggests that this association can also act positively by providing a ‘landmark’ for Z-ring assembly between the replicated chromosomes. Although normally this process appears to be relatively weak, it plays a more prominent role in cells lacking MinC and SlmA [51]. Interestingly, since the *Ter* appears to leave mid-cell just prior to constriction, might this process also communicate the completion of chromosome segregation? [47^•^].

A third distinct possibility is that DNA-translocases present in the division machinery such as FtsK/SpoIIIE proteins might clear the DNA from beneath the closing septum [52, 53]. In *Streptococcus pneumoniae* this process may play a more prominent role, especially given the small volume of these cells and the observation that the Z-ring appears to assemble on top of unsegregated nucleoids [34, 54, 55]. However, while DNA translocation provides a valuable ‘fail-safe’ mechanism, another level of redundancy would seem desirable, particularly since results in other organisms demonstrate that DNA translocases are not always 100% effective [22••, 23••, 35•].

## Positive regulation of division site selection

In contrast to the well-studied systems that negatively regulate division site selection, it has been proposed that additional mechanisms might exist that act positively, for example, by contributing an essential division component or by counteracting an inhibitor [37•, 56]. Rodrigues and Harry [37^•^] recently showed that in a *B. subtilis min noc* background the frequency of Z-ring assembly was both dramatically reduced and subject to a significant delay. Nevertheless, in a small population of cells there was a substantial preference for Z-ring assembly at mid-cell, independently of either the Min system or the nucleoid [37^•^], which the authors propose might result from unknown factors that actively identify the division site [37^•^]. Currently, however, only two examples of positively acting systems have been reported in bacteria.

In *Streptomyces* spp., which are multi-nucleate filamentous bacteria that produce long chains of spores, FtsZ initially assembles extended ‘spiral’ structures on top of the nucleoids during sporulation, before forming a ‘ladder’ of Z-rings between the segregated nucleoids [57]. Remarkably, SsgA directs the localisation of a membrane-associated protein, SsgB, into these inter-nucleoid spaces where it recruits FtsZ, and possibly enhances Z-ring assembly [58^••^]. But how does SsgA direct its partner to the correct site? Willemse *et al*. [58^••^] highlight that in a filamentous bacterium where the cell poles are distant the nucleoid presents an ideal candidate to control this critical task. A comparable mechanism is used by *Schizosaccharomyces pombe*, whereby the nucleus restricts the localisation of Mid1p, which acts positively to position the division site [59, 60]. More recently, the ParA-like protein PomZ (*Po*sitioning at *m*idcell of Fts*Z*) was found to positively regulate Z-ring positioning in *Myxococcus xanthus*, although it probably acts indirectly since a direct interaction with FtsZ could not be established [61]. Interestingly, PomZ first localises over the nucleoid, before moving to mid-cell ahead of FtsZ, raising the possibility that this might be triggered by the completion of chromosome replication/segregation. Furthermore, abnormal cell divisions in Δ*pomZ* cells never occurred over the nucleoids, indicating that *M. xanthus* probably contains a nucleoid occlusion system [61].

## A role for ParABS systems?

Although *Caulobacter crescentus* lacks obvious homologues of MinCD or nucleoid occlusion factors, an alternative mechanism has been identified that combines aspects of both systems. MipZ (*mi*d-cell *p*ositioning of Fts*Z*), a divergent ParA/MinD family ATPase, is essential for the correct placement of the division site [62^••^]. It forms ATP-dependent dimers that interact with and perturb FtsZ polymers by stimulating their GTPase activity [62^••^]. Strikingly, dimer formation is stimulated by ParB, which is localised at the cell poles with the origin [63]. Consequently, MipZ dimers emanate outwards from the cell poles on DNA such that their lowest concentration is towards mid-cell (Figure 1c) [62••, 63]. Nevertheless, since cell division apparently initiates with the chromosome still present at mid-cell, other factors are likely to be involved [64]. Interestingly, ParA-like proteins may also play a role in division site selection in *Corynebacterineae* (see for [65] in-depth review). In *Corynebacterium glutamicum* PldP, an orphan *P*arA-*l*ike *d*ivision *p*rotein, localises over the nucleoid and at the nascent division site [66]. Since null mutants exhibit a division defect, PldP might help to link chromosome segregation and cell division in *C. glutamicum* [66, 67]. Similarly, a ParA-like protein also seems to have a division specific role in *Mycobacterium smegmatis*, but whether it acts directly remains unclear [68].

## Concluding remarks

The identification of Noc and SlmA provided a molecular basis for nucleoid occlusion and considerable progress has been made in the last decade in understanding the mechanisms by which these factors act. While the primary role of nucleoid occlusion is almost certainly to prevent catastrophic guillotining of the genetic material, in *B. subtilis* and *E. coli*, Noc and SlmA also play integral roles in helping to specify the spatial and temporal organisation of division. Noc may also play a role in restricting division to one plane in *S. aureus*. Nonetheless, it is clear that in some bacteria other factors may act to protect the DNA at later stages of division, for example, DNA translocases. One important area of future research will be how nucleoid occlusion varies with growth rate, particularly since during slow growth, in *E. coli* at least, the chromosomes appear to segregate as cell division initiates [69]. Another open question is whether nucleoid occlusion is active in bacteria with fundamentally different modes of growth and division, for example, symbiotic bacteria that grow in width [70] or in bacteria that divide independently of FtsZ [71, 72]. Similarly, how does nucleoid occlusion differ in bacteria such as *V. cholerae* that have more than one chromosome? As the range of organisms studied and the development of novel genetic and cell-biology tools continues to rapidly expand, we anticipate new insights into how diverse organisms tackle this fundamental problem.

## References and recommended reading

Papers of particular interest, published within the period of review, have been highlighted as:

• of special interest
•• of outstanding interest

## Figures and Tables

Images of Noc are adapted from [24^••^]. Images of SlmA are courtesy of Hongbaek Cho and Nick Peters.
